# A chemical energy approach of avascular tumor growth: multiscale modeling and qualitative results

**DOI:** 10.1186/s40064-015-1417-5

**Published:** 2015-11-02

**Authors:** Pantelis Ampatzoglou, George Dassios, Maria Hadjinicolaou, Helen P. Kourea, Michael N. Vrahatis

**Affiliations:** School of Science and Technology, Hellenic Open University, Sachtouri 11, 26222 Patras, Greece; Department of Chemical Engineering, University of Patras, Patras, Greece; Medical School, University of Patras, Patras, Greece; Department of Mathematics, University of Patras, Patras, Greece

**Keywords:** Breast cancer, Tumor growth, Avascular, ATP, Multi-scale model, Primary 92C05, 92D25, 92C15

## Abstract

In the present manuscript we propose a lattice free multiscale model for avascular tumor growth that takes into account the biochemical environment, mitosis, necrosis, cellular signaling and cellular mechanics. This model extends analogous approaches by assuming a function that incorporates the biochemical energy level of the tumor cells and a mechanism that simulates the behavior of cancer stem cells. Numerical simulations of the model are used to investigate the morphology of the tumor at the avascular phase. The obtained results show similar characteristics with those observed in clinical data in the case of the Ductal Carcinoma In Situ (DCIS) of the breast.

## Background

In 2014 in the US, there is an estimation of 1,665,540 new diagnosed cancer cases and 585,720 deaths. Cancer maintains its ranking as the second most common cause of death in the developed and developing countries, accounting for nearly a quarter of deaths (Siegel et al. 2015). Among others, these statistics make cancer the most urgently investigated disease in today’s research efforts.

### Cancer description

Neoplasm or tumor is an independently growing mass of abnormal cells. The tumors that remain localized, cannot metastasize to other organs and are therefore innocent called benign tumors. Malignant tumors, also called cancers, are locally destructive neoplasms with the potential for distant spread, thus causing death (Goldschmidt and Chief 2013). Cancers are classified by the tissue from which they arise and by the type of cells involved. For example, leukemia is a cancer of circulating white blood cells, sarcoma is a type of cancer arising from tissues of mesenchymal derivation such as adipose or connective tissues, and carcinoma is a cancer originating from epithelial cells. The epithelial cells, are cells closely interconnected in such a way as to form acinar or glandular structures, or to line cavities or tubular organs. Such examples are the cells forming the respiratory or gastrointestinal tract or solid organs such as the breast, the pancreas and the prostate gland, among several others. Until recently, the classical model of carcinogenesis stated that cancer starts when a cell inside a tissue is subjected to DNA mutations that change its phenotype to cancerous. A primary tumor, which is a tumor in its original site of occurrence, is usually traced to a single mutated cell, from which over a period of time, a colony of cells is formed. Progeny of cancer cells reproduce at a faster rate than normal cells forming a colony. In cancers of epithelial origin, namely carcinomas, this colony is delimited by a basement membrane that isolates the neoplastic cells. At this phase, the tumor is called carcinoma in situ and has no metastatic potential. Once the neoplastic cells acquire additional abilities to break out of the basement membrane, than they invade the surrounding extracellular matrix (ECM) and the tumor is called invasive carcinoma and can find access to vessels and produce metastases. Cancers can grow up to 1–2 mm$^3$ by obtaining oxygen and nutrients through the existing vasculature (Folkman 1990). During this stage the tumor is said to be avascular. Invasive carcinoma cells can find access to old and newly formed blood vessels. Once the tumor cells enter the blood vessels they can be transported via the circulation to other organs, where they can exit from the circulation, engraft in the new environment and start to grow again to produce a metastatic or secondary tumor. In this manuscript we consider only the early stages of cancer development, while the tumor remains in an avascular state. Traditionally the cause of cancer is considered being the fact that DNA replication and repair is not a 100% accurate process, and cancers emerge as a result of the many gene mutations accumulating in the human body over a person's lifetime. There is evidence that a single abnormal cell, which gives rise to a tumor, has risen through a number of genetic alterations, or epigenetic mutations; the latter means a change of gene expression as a result of blocking of gene promoters. The two main ways by which genes can become oncogenic are: (1) a stimulating gene becomes hyperactive, or upregulated; such an abnormal gene is called oncogene; and (2) an inhibitory gene becomes inactive, or downregulated; it is called a tumor suppressor gene, an example being the p53 gene which controls the progression of the cell cycle. In order to continue to grow, the tumor requires new sources of nutrients. It does it by secreting chemicals called tumor growth factors, which stimulate the formation of new blood vessels, attracting them into the tumor. This is the process of angiogenesis; a tumor which has developed beyond this stage is said to be vascularized. Further on, the progeny of cancer cells have a higher probability of mutations that lead to increased cancer aggressiveness over time. Breakthroughs in medical science led to the discovery that cancer is initiated not only by the mutation of a cell’s DNA but rather by the mutation of a stem cell’s DNA (Reya et al. 2001; Jordan et al. 2006). This mutated stem cell now named cancer stem cell travels inside the tissue like normal stem cells, producing cancer cells.

### Modeling

Eykhoff in 1974 defined a mathematical model as a ‘representation of the essential aspects of a system which presents knowledge of that system in usable form’ (Åström and Eykhoff 1971). Considering cancer as a multiparametric complex dynamical system, mathematical modeling aims to provide a better understanding of the mechanisms causing the tumor growth and contribute to the diagnosis, therapy and prevention. Through mathematical methods, the empirical and qualitative observations and experimental data can be explained and integrated to realistic models that may also serve as a indispensable ‘non-invasive tools’, for either assessing a treatment (therapy) or for understanding the biological rules of growth. Numerous mathematical models have been developed in the last 50  years for the study of tumor growth, in different phases (Lowengrub et al. 2010). Most of these mathematical approaches fall initially into two general categories, the continuum and the discrete; based on the assumptions made for the tumor tissue. Continuum models represent the main category of models employed in this field, (see for example Murray 2003; Chaplain and Lolas 2005), where the tumor is considered to be homogeneous and continuous averaging out the effects of individual cells. Its growth is described through the evolution of its boundary. Exemplary efforts of continuum models for cancer growth are those provided by Byrne and Chaplain (1996) and Byrne et al. (2003) where the proposed models describe the evolution of the boundary of an avascular solid tumor driven by nutrient supplied by the extracellular matrix. Cellular movement has been studied in (Macklin and Lowengrub 2007; Greenspan 1976; Friedman 2007; Byrne and Matthews 2002; Chaplain and Sleeman 1993). Continuum cancer models are focusing on the evolution of the densities of cells (abnormal, normal, or dead), and the evolution of boundaries of the tumor regions which are due mainly to changes of the concentrations of biochemical species, are described by differential equations. Their approach is based on the principles of continuum mechanics. Some of these models use only ordinary differential equations (ODEs) (Greenspan 1972; Andersen et al. 2005), overpassing the spatial heterogeneity of tumor growth. Otherwise Partial Differential Equations (PDEs) of reaction diffusion type take into account spatial effects and also may describe the time evolution of the tumor region. Since a deterministic approach neglects random influences on the growth process, stochastic differential equations can be regarded as more adequate models for the development of a population (Rosenkranz 1985). Additionally one of the methods that is applied to describe tumor growth is the dynamic scaling of interfaces based on the work given by Brú et al. (2003). There is strong experimental evidence that the fluctuations of tumor region boundaries show temporospatial behavior, that contain the characteristics of self-affinity. The interface fluctuations seem to evolve according to power laws. This approach is used as a starting point to describe the evolution of tumor boundaries through the use of stochastic partial differential equations.

Discrete models on the other hand consist a separate category, where the behavior of the tumor is determined by the interaction between individual cells and the microenvironment both. Recently, discrete-numerical models (Swanson et al. 2002; Clatz et al. 2005) are given much attention due to the increase in computer power that is available nowadays. In these numerical models the biological processes are described using mathematical tools, available from numerical analysis methods. Special reference is due to the Cellular Potts Model (CPM) also known as extended large-q Potts model or Glazier and Graner model. The CPM is a lattice-based computational modeling method to simulate the collective behavior of cellular structures with notable applications on cancer modeling (Graner and Glazier 1992; Ghaemi and Shahrokhi 2006; Turner and Sherratt 2002; Stott et al. 1999). In the CPM the process of the simulation progresses by updating the cell lattice one pixel at a time based on a set of stochastic rules. CPM can be thought of as an agent based generalized cellular automaton method in which cell agents interact through precise methods. Hybrid models employ differed methods from both continuum and discrete mathematics in order to archive a better modeling approach of the studied phenomenon. In the case of hybrid tumor modeling, continuum methods are being used mainly to model the tumor on a macroscopic scale while discrete functions are applied mainly to the microscopic scale. Multiphase modeling is used in modeling physical phenomena that are described by two-or-more liquids that flow on different phases. Each of the phases is considered to have a separately defined volume fraction and velocity field. In the case of cancer multiphase modeling, the tumor is described as two or three separate liquids, depending on the assumptions of each approach. These liquids are correlated with corresponding types of tumor cells and are presumed to flow with different velocity fields, thus simulating the evolution of the various tumor regions in time (Sciumè et al. 2013). Multiscale approach is employed in order to solve problems which have important features at multiple scales. Multiscale models may serve as theoretical tools but also allow for a deeper understanding of the underlined biological system. These models incorporate biological mechanisms that refer to intracellular, cellular or extracellular scale. While employing multiscale modeling several restrictions have to be taken under consideration, the most important of which is scale linking (de Pablo 2011; Steinhauser 2008). A detailed review of the various proposed mathematical models for tumor growth can be found in the work of Lowengrub et al. (2010).

## Results and discussion

In the present manuscript we propose a hybrid multiscale model for avascular tumor growth. At the tissue scale, the concentrations of the encountered biochemical species are described through diffusion equations, while at the cellular scale cellular life is modeled as an cellular automaton. At this scale we introduce the concept of a function that encounters the chemical energy level of each cell in ATPs. The existence of this function in the model affects the behavior of the cells and consequentially determines the morphology of the tumor. At the intracellular scale, stochastic methods describe the behavior of the cellular organs.

### Results

Based on the configuration and methodology explicitly presented in the following sections, we demonstrate our early results (of 30 simulations) that describe the behavior of a tumor. The qualitative characteristics of the proposed model are in accordance to other mathematical models of avascular tumor growth, while in addition, qualitative information of the tumor morphology is gained, which is the formation of necrotic islands in the center region of the tumor. The obtained results coincide with observed clinical data. In more detail the following figures depict a series of cross sections of a simulated three dimensional tumor at different time iterations, where the simulated cells are shown as circles.

**Fig. 1:**
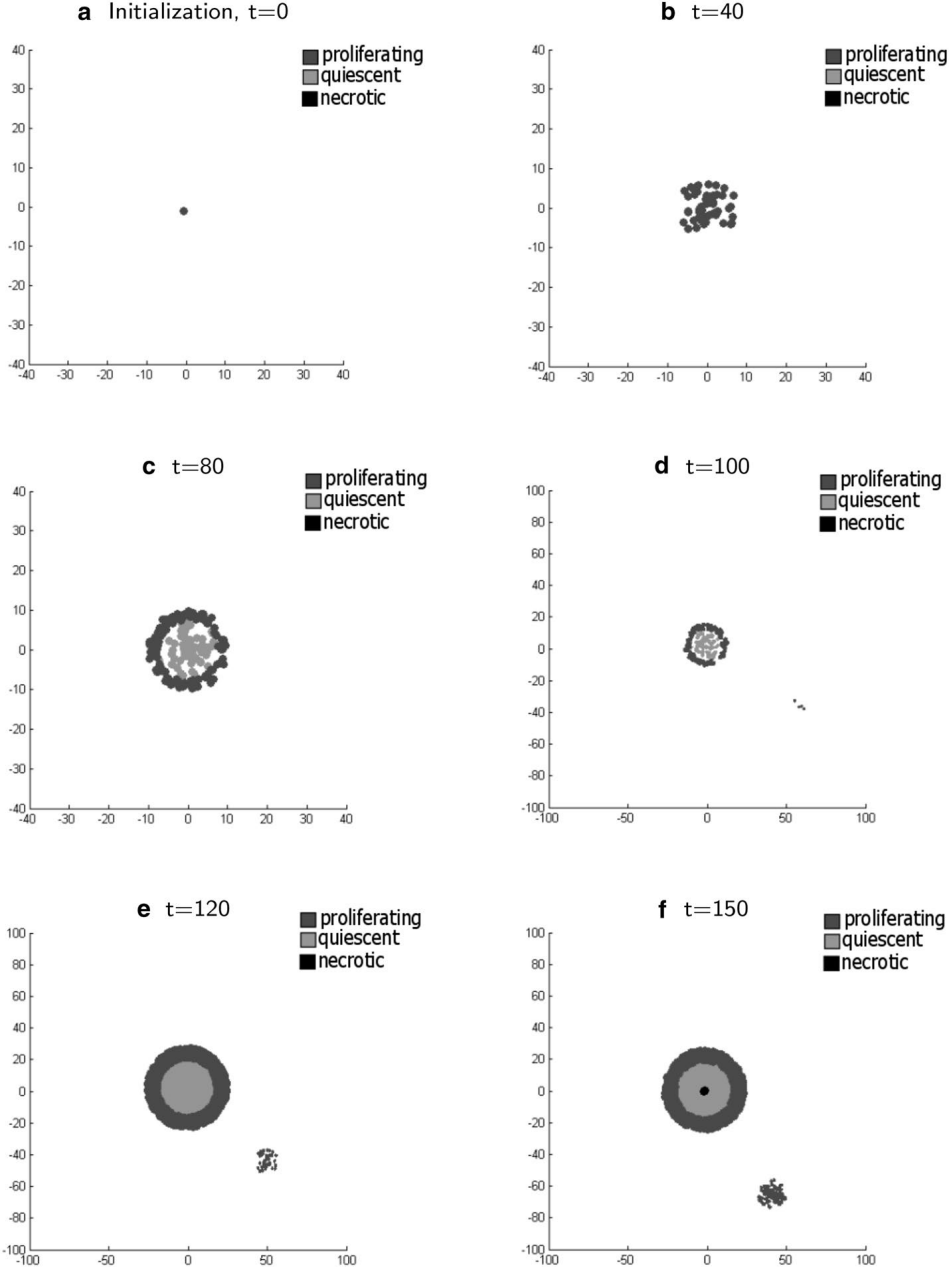
Time lapse of a cross sections from a characteristic three dimensional tumor simulation. Initialization and progress of cancer tumor growth, depicting the formation of regions and secondary tumors

Figure 1a shows initialization of the model and the first cancer cell that is added. Until an initial time period of 46 intervals, the population of the proliferating cancer cells keeps following an exponential growth which is attributed to the extremely rich biochemical environment which allows for high values of the health function (11) of all the cells and thus at a high proliferating rate. This can be observed in Fig. 1b.

After the 52 time iteration, we notice that the amount of nutrient doesn’t suffice to allow the entire population of cancer cells to remain proliferating and so we observe a quiescent region appearing in the center of the tumor. This results in reduction of the growth rate of the cancer cell population. Such behavior is depicted in Fig. 1c. As simulation time progresses, we observe that the cancer stem cell produced a new proliferating cancer cell that in turn, starts an exponentially growing second tumor. Such behavior is depicted in Fig. 1d. As time progresses, we notice that the growth of the population of the second tumor, even though it remains exponential, it is of a lower rate than the one of the original tumor. This behavior is due to the fact that the total amount of nutrient inflow, through the tissue remains constant. Therefore the second tumor behaves as if it grows in a poorer biochemical environment. This is shown in Fig. 1e.

In Fig. 1f we observe the existence of a necrotic region, which appears at the center of the first tumor. This is due to the lack of sufficient amount of nutrients in the simulated area. Thus the tumor can not sustain the entire cell population and cells that exist in the domain with the poorest biochemical environment undergo necrosis.

**Fig. 2:**
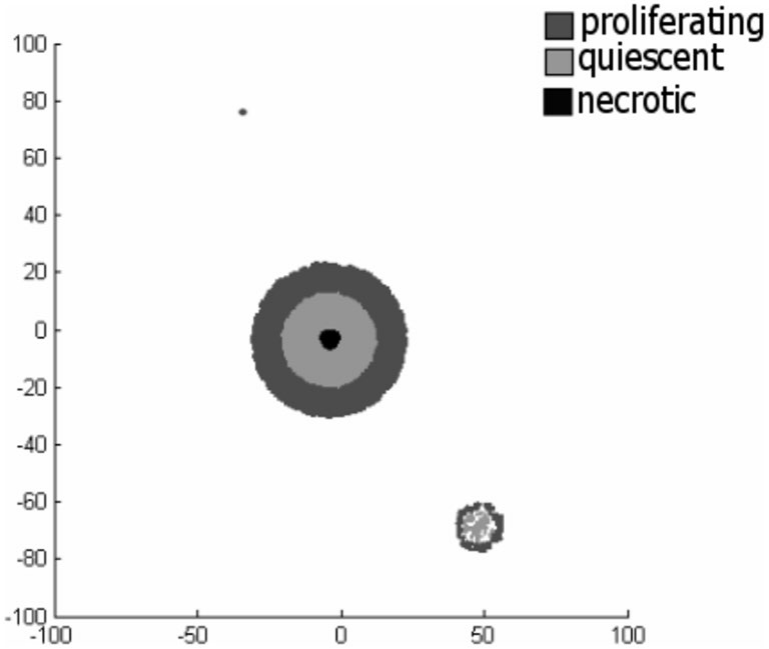
Appearance of quiescent region at the center of the second tumor as well as expansion of the necrotic region of the first tumor

**Fig. 3:**
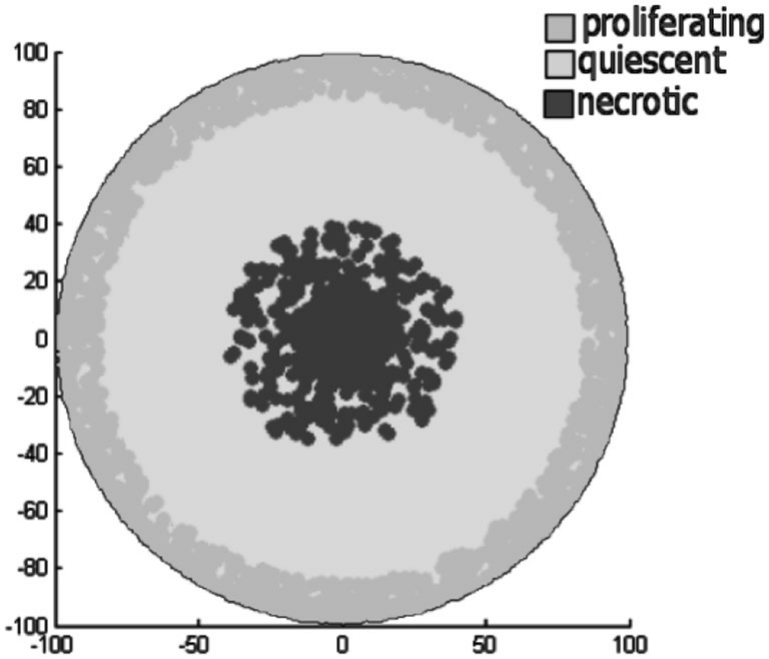
Two-dimensional cross-section of a simulated three-dimension tumor. The figure demonstrates a two-dimensional cross-section of a simulated three-dimension tumor

In Fig. 2 we first show that the cancer stem cell introduces a new cancer cell to the model. Secondly we observe that although the area of the first tumor remains intact allowing small fluctuations we observe new necrotic cells to appear. This is attributed to the degradation of the biochemical environment in the region from the growth inhibitors. Finally, we detect that the second tumor shows increased thickness of the proliferating area which is closer to the boundary of the simulated area. This is due to the inflow of nutrition from the extracellular environment that allows for richer biochemical surroundings on that side of the tumor. Also focusing in to the quiescent region of the tumor depicted in Fig. 3, the appearance of agglomerations of necrotic cells is evident. These agglomerations provide a better insight of the morphology of a tumor.

### Model validity and generalization

**Fig. 4:**
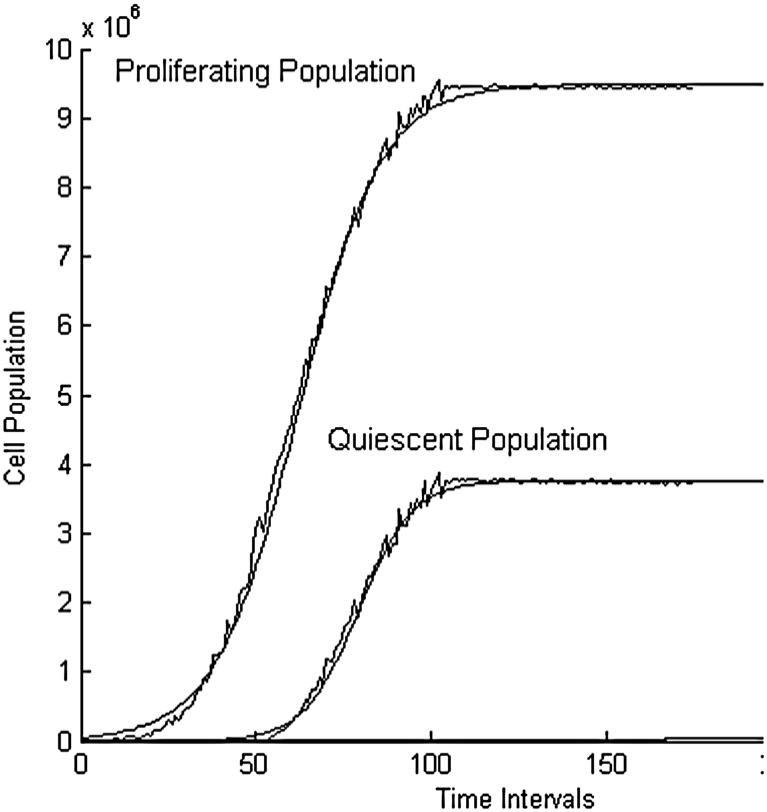
Population of cancer cells over time. The *dashed lines* represent the number or quiescent and proliferating cells in the simulated area at time iteration t, and the *smooth lines* represent the data fit estimations of the two cellular population growths

Studying the evolution of cancer cell population over time iterations, the proposed model produces various growth patterns that all converge to a maximum value of each type of proliferating and quiescent cells. In the following figure we show the evolution of simulated tumor cell populations over time. The lines in Fig. 4 represent the evolution of the population of each category of cancer cells with green, blue and black representing respectively quiescent, proliferating and necrotic cells.

In the simulations made under the aforementioned assumptions, the proposed model shows that the evolution of the population of quiescent cells over time *Q*(*t*) is described from the following function:

1 $$\begin{aligned} Q(t) = \frac{9.5322E+06}{1 + e^{-0.0826 \left( t- 61.2127\right) }} \end{aligned}$$

while the evolution of the population of the proliferating cells over time *P*(*t*) is described from the following function:

2 $$\begin{aligned} P(t) = \frac{3.9289E+06}{1 + e^{-0.1193 \left( t- 79.0278\right) }} \end{aligned}$$

From (2) and (1), we can extrapolate that the total population of living cells of the simulations is 1.3461E+07. This value depends on the imposed boundary assumptions. These estimation functions cross validated against the produced simulation data using the Leave-p-out methodology. Aiming to verify qualitatively the results obtained through the model, we compare them with those derived by classical population models.

### Reduction to known results

**Fig. 5:**
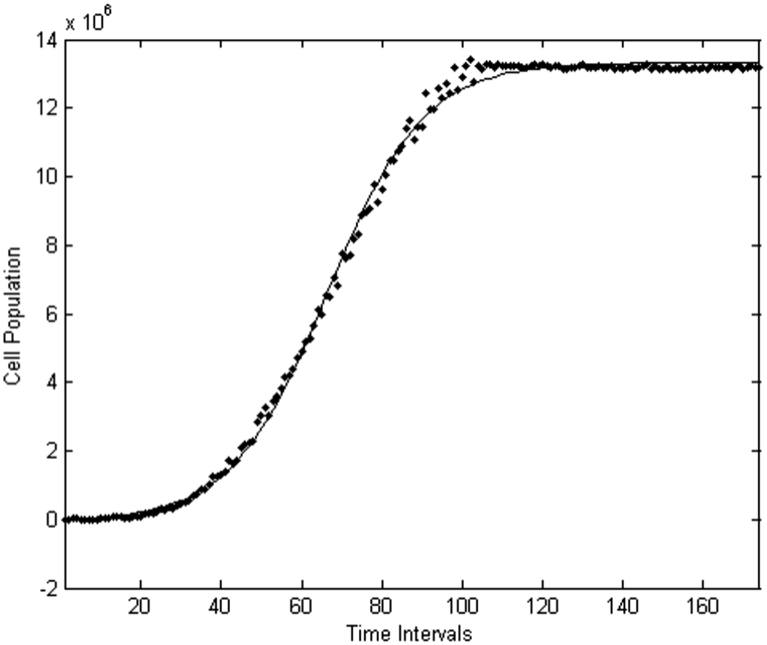
Average tumor cell population over time. The points represent the average over multiple simulations number of cells at time iteration t, and the *continuous line* represents logistic fit of the average cell population at time iteration t in the domain $\varOmega$

In order to verify quantitatively the obtained results, the method of data fiting is employed. Given the obtained results from the simulations performed and applying various sigmoid data fitting functions, such as Gompertz, Logistic, Hill, Chapman, Boltzmann and Don Levin, the best fit is produced by the Logistic function (3) with R-squared: 0.998563718067 and is depicted in Fig. 5.

The general form of the logistic function is:

3 $$\begin{aligned} \frac{dN}{qt}=rN\left( 1-\frac{N}{K}\right) \end{aligned}$$

where N corresponds to the population of living cells, K is the carrying capacity of cell population given the biochemical environment and the ECM, r is the growth rate and $N_{0}$ is the cell population at the beginning of the simulation. The solution of Eq. (3) is:

4 $$\begin{aligned} N(t)=\frac{KN_0 e^{rt}}{K+N_{0} (e^{rt}-1)} \end{aligned}$$

Now given that N represents the population of simulated cancer cells, and thus is a positive integer, and in order to produce a simpler mathematical model for the simulations with discreet time, the following hazard function (Gillespie 1976) is applied on the Logistic equation.

5 $$\begin{aligned} h(N,r,K)=rN\left(1- \frac{N}{K}\right) \end{aligned}$$

Starting from time t = 0 and a single cancer cell at that time, we can calculate the time intervals until the next mitosis by using the values of the distribution:

6 $$\begin{aligned} \delta t=e^{h(N,r,K)} \end{aligned}$$

**Fig. 6:**
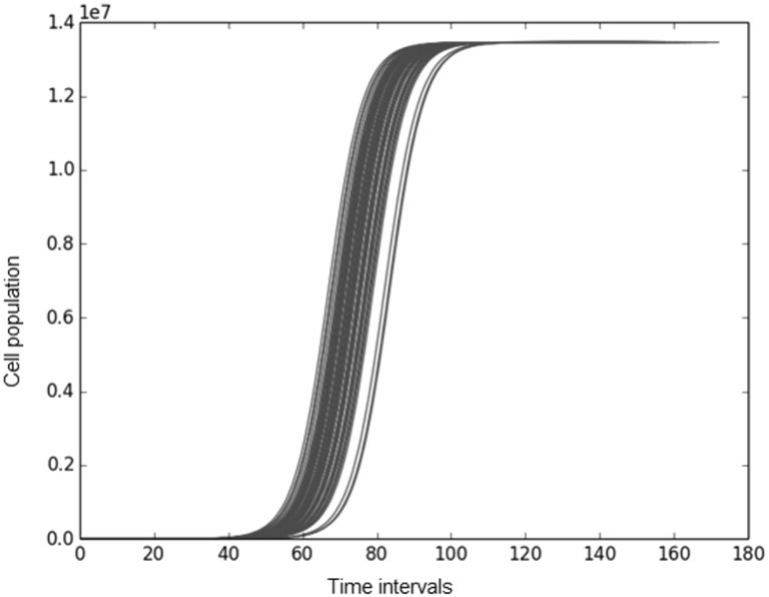
Tumor cells population growth. The *lines* represent the hazard simulations of population growth for the tumor cells. As computer model time t progresses by $t = t + \delta t$ the population of cancer cells increases until the carrying capacity K is reached. In this figure such a simulation is shown with $K=1.3461E+07$, $r=0,21583949$ and $N_{0}=1$. The vertical axis represents the population of cancer cells, while the horizontal axis represents time iterations

Thus, cancer cell population growth over time can be calculated as demonstrated in the following Fig. 6. We depict in this figure that for time t = 0 a single cancer cell exists in the simulation environment.

### Discussion

Improving our previous work on the proposed model (Ampatzoglou and Hadjinicolaou 2013) we extended the model by implementing a mechanism for the induction of cancer cells from cancer stem cells. In medical literature, these CSC are considered to travel inside the tissue and spore at times new cancer cells. This expansion is included in the proposed model by a mechanism that allows for a CSC to travel freely inside the simulated area and randomly produce daughters that are cancer cells which can produce new tumor ‘islands’. This expansion of the model derives simulation results that are consistent with the previously proposed model and are in accordance with the observations of in-vivo cancer tumors that usually show a non-well-formed and consistent cancer tumor, but rather multiple and fluctuated tumors that appear in the form of cancer agglomerations within the tissue. Moreover, given the finite rate of inflow of biochemical factors inside the tumor, we observe a competition for nourishment between the different tumor islands. Simulations show that the new tumor islands that are introduced to the model from the cancer stem cell deprive already existing tumors from nutrients, thus forcing them to reduce the number of cells.

It is well documented both in-vino and in-vitro, that avascular carcinomas can show complex structures that deviate from the standard spheroidal patterns (see for example Bredel-Geissler et al. 1992; Byrne and Matthews 2002). Similar morphologic characteristics are evident in the proposed model mainly in the development of the necrotic region where the necrotic region is not a spherical or a symmetric continuous domain, but rather is divided in two sub-regions. One in the center that is spherical and is occupied solely from necrotic cells and a second area that is occupied from both quiescent and necrotic cells with the later forming complex clusters and agglomerations. Similar formations documented appear in many types of human tumors such as the case of human prostate cancer (Hedlund et al. 1999) and seems to be in accordance with real data obtained in the case of the Ductal Carcinoma In-Situ of the breast published from Fonseca et al. (1997).

## Conclusions

We propose a lattice free multiscale model, that describes avascular tumor growth through a chemical energy vantage point, using the ATP molecules as a quantification approach to reveal cellular dynamics. The proposed health function offers greater resolution and insights to cellular dynamics with respect to small time intervals; in contrast to other tumor models where such effects are averaged. Tumor cells are persevered as incompressible bodies that react to the cellular environment both biochemicaly and mechanicaly. The biochemical environment is described by the concentrations of biochemical species, that propagate through the studied area through diffusion. The values of the concentrations of these species are calculated using finite element methodology. Cellular movement is implemented as a result of both chemotaxis and a spring based cellular adhesion hypothesis. Estimations made for various parameters of the model are explained. The model requites calibration in order to produce results that are better approaches to observed tumor behavior.

The model predicts (1) avascular tumors that are growing within a circular or spherical extracellular environment are likely to reach and oscillate around equilibrium. (2) The population of tumor cells depends on the amount of nutrition that it is provided to the tumor by the host tissue through the ECM. This is a result of the implemented chemical energy approach that restricts the population of cells that can be sustained from the nutrients that are offered to the tumor by the ECM. (3) The model demonstrates complex formations of necrotic regions scattered around the center of the tumor as shown in Fig. 3.

**Fig. 7:**
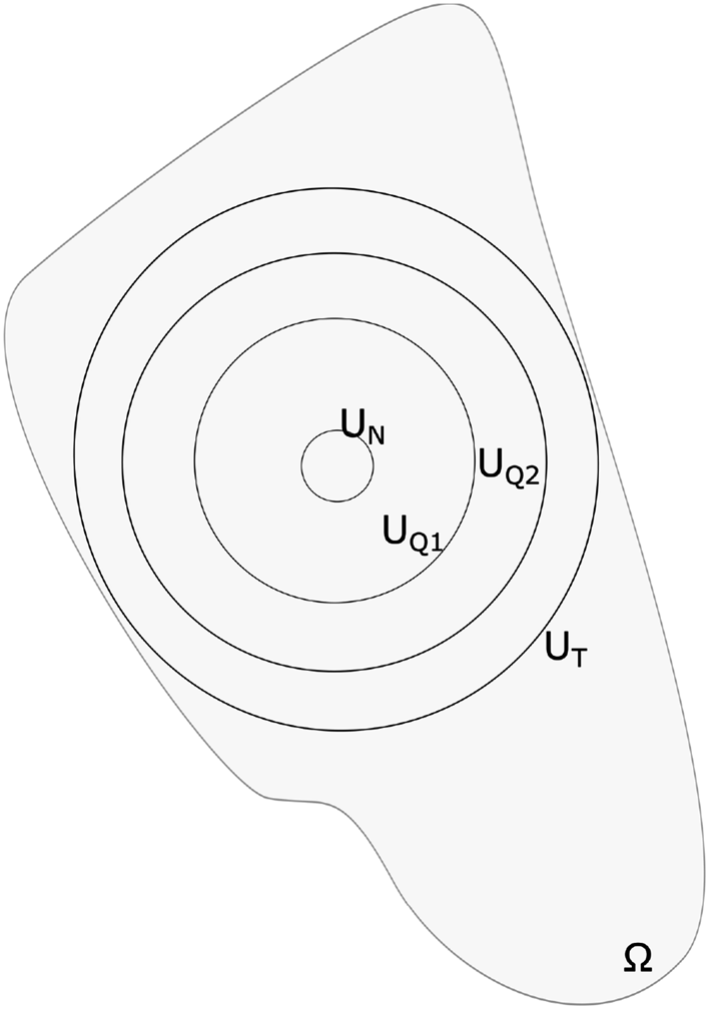
Tumor regions. The different regions produced by the model inside the studied boundary $\varOmega$

Figure 7 presents the different regions of the proposed model. The tumor grows inside the studied area $\varOmega$ and shows the aforementioned regions with all tumor cells inside the $U_{T}$ boundary. The innermost region of the simulated tumors $\varOmega _{N}$ exists inside the boundary noted as $U_{N}$. In this area all cells are necrotic as a result of low concentrations of oxygen and glucose that are incapable to provide enough energy to the cells to remain alive. Further the model shows a region between the boundaries $U_{Q1}$ and $U_{Q2}$, where all cells remain in a quiescent state. In this area noted as $\varOmega _{Q}$ the biochemical environment can support cellular life but is unable to provide the high amount of nutrients and oxygen required by cells to proliferate. Also, the domain between $U_{Q2}$ and $U_{T}$ is occupied by both proliferating and quiescent cells. Both these types of cells appear in this region because once a mother cell reaches mitosis, its chemical energy is equally divided to the two daughter cells. Thus each daughter cell has a chemical energy level or health level that is characteristic of quiescent cells. In the area between the $U_{N}$ and $U_{Q1}$ boundaries, defined by $\varOmega _{A}$ a mixture of both quiescent and necrotic cells exists. In this area formations or agglomerations of necrotic cells appear as a result of the model's considerations, assumptions and is not imposed.

Region $\varOmega _{A}$ is an innovation when compared with other mathematical models of cancer tumor development for the following reasons: such a region is not predicted by other models; or is observed under a forced behavior as a direct result or border fluctuations. In the proposed model no such rule or assumption is made that forces the creation of such artifacts inside the tumor region. Rather, they are produced as a result of the health level function that allows for greater insights to cellular behavior.

## Methods

The proposed model is a multiscale model for tumor growth. The tumor is presumed to consist of cancer cells that develop and evolve freely inside the studied area. These cells in accordance with other cancer models belong to three categories that are explained in the following section. The behavior of the tumor as a whole as well as the behavior of each participating cell individually, is an implicit result of the biochemical environment in which the cells exist. The participating biochemical species are: oxygen, glucose, waste, growth factors and growth inhibitors. The later three are abstract biochemicals that include a number of different factors and proteins of cellular life affecting the tumor similarly. For example the generic term waste envelops the total of metabolic end products that cells produce such as pyruvate, lactate, alanine, proline, aspartate, and citrate (Lanks and Li 1988). Further, we presume that inside the studied area, cancer stem cells (CSC) exist, that are able to randomly produce new cancer cells inside the ECM every 70 time intervals.

### Formulation of the problem

**Fig. 8:**
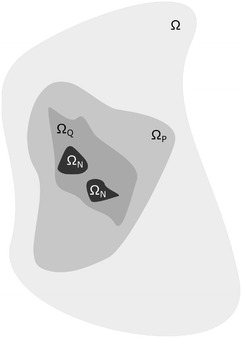
Tumor regions. The studied area $\varOmega$ including areas occupied by proliferating ($\varOmega _{P}$), quiescent ($\varOmega _{Q}$) and necrotic ($\varOmega _{N}$) cells

For the purpose of this model we assume that the tumor starts and develops over time in the tumor domain $\varOmega$. $\partial \varOmega$ is the boundary between the tumor tissue and the host tissue as show in Fig. 8. The domain $\varOmega$ is occupied by the extracellular fluid (ECF) that acts as a substrate where all the biochemical factors ‘travel’. ECF corresponds to all body fluids outside the cells. Further on, we assume that the evolution of cancer cells is a result of the biochemical environment of each cell. The biochemical species that are considered to constitute the aforementioned environment oxygen, glucose, waste, growth factors and growth inhibitors. Each of these species (1) participates in the evolution of the tumor according to its concentration $C_{i}(u,t)$.

7 $$\begin{aligned} C_{i}(x,t)=C_{i} constant, x \in \partial \varOmega \end{aligned}$$

Also an initial state at time $t=t_{0}$ is imposed for the domain $\varOmega$ where for each biochemical species concentration an initial value is assumed:

8 $$\begin{aligned} C_{i}(x,t_{0})=C_{i} constant, x \in \varOmega \end{aligned}$$

**Fig. 9:**
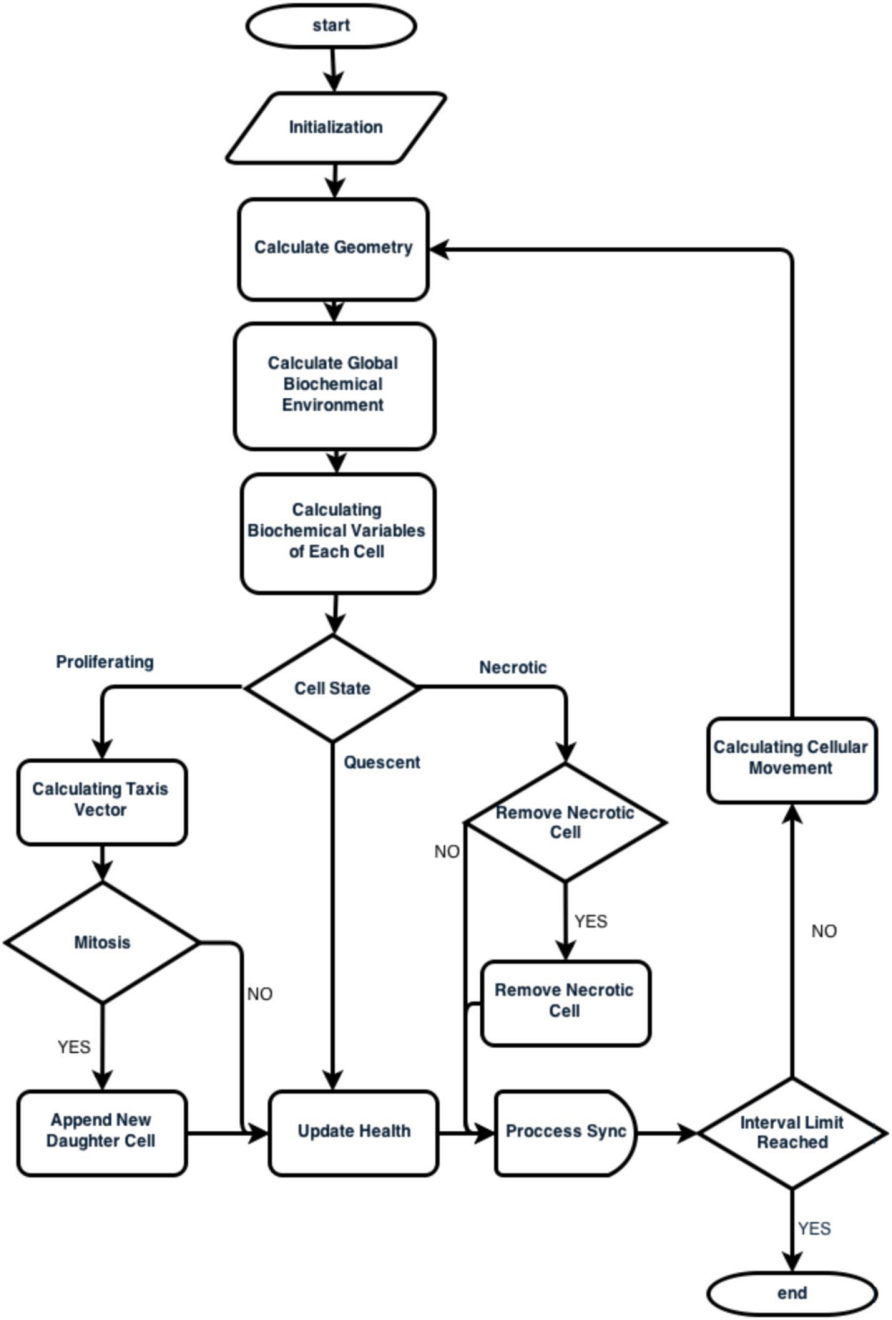
Model flowchart

Further on, in accordance with classical multicellular spheroid models (Greenspan 1972), we assume that inside domain $\varOmega$, there can be cancerous cells that according to their state can be divided in three distinct categories: proliferating (cells in the G1, S, G2 and M phases); quiescent cells, representing cells in the G0 phase; or necrotic cells. These cells abide to adhesion mechanisms, try to move according to chemotaxis laws and undergo mitosis under a specific rule framework described in the following sections. These cancerous cells may be of any geometric shape and size and can move freely inside the domain $\varOmega$. This configuration allow without enforcing, the creation of regions occupied by proliferating, quiescent and necrotic cells. These areas in Fig. 8 are noted by $\varOmega _{P}$, $\varOmega _{Q}$ and $\varOmega _{N}$ respectively. The flowchart of the model is presented in Fig. 9.

### Tissue scale

In the proposed model, the biochemical environment of the cells is described through the concentrations of five biochemical species, namely oxygen, glucose, waste, growth factors and inhibitor factors. All of these biochemical substances are provided either by the tumor environment, (oxygen and glucose) and are consumed by the cells or they are produced by the cells and removed through the extracellular tissue (waste, growth factors and inhibitor factors). Propagation of these biochemical species through the domain $\varOmega$ is modeled by diffusion equations with prescribed diffusion constants based on experimental data given by Jiang et al. (2005) as shown on Table 1.

**Table 1:** Diffusion constants for biochemical species (Jiang et al. 2005)

	Oxygen	Glucose	Waste	Growth	Inhibitor
Diffusion constant	$5.94\times 10^{-2}$	$1.52\times 10^{-3}$	$2.124\times 10^{-3}$	$10^{-6}$	$10^{-6}$
Unit	($\text{cm}^{2}/\text{h}$)				

A Dirichlet boundary condition is applied at the boundary $\partial \varOmega$ expressing the assumption of a constant level of concentrations for all these species at the $\varOmega ^{-}$ and therefore at the boundary $\partial \varOmega$. When setting up a mathematical model of a biological process, it is very important to determine the numerical value of the parameters, because biological processes are typically valid only within a limited range of parameters. Further on, each biochemical species is considered to be produced or consumed by each cell independently according to its state. The equation that is used to describe the diffusion for each of these biochemical species for each cell and thus allows the calculation of the concentration of each biochemical factor at each location inside the model is:

9 $$\begin{aligned} \frac{\partial {C}_{i}}{\partial t}+\nabla \cdot \left( -D_{i}\nabla C_{i}\right) = Q_{i,j} \end{aligned}$$

where i = 1,2,…,5 corresponding to the oxygen glucose.... respectively.

- $C_{i}$: concentration of biochemical specie ‘i’
- t: time.
- $D_{i}$: diffusion coefficient of biochemical factor ‘i’
- $Q_{i,j}$: rate of production-consumption of biochemical factor ‘i’ dependent on the state of cell ‘j’ the values of which given in Table 2.

**Table 2:** Metabolic rates for the biochemical species for proliferating, quiescent and necrotic cancer cells (Jiang et al. 2005)

State	Oxygen	Glucose	Waste	Growth	Inhibitor
Proliferating	108	162	240	1	0
Quiescent	50	80	110	0.5	1
Necrotic	0	0	0	0	2
Units	mM/h/cm^3^			%/h/cm^3^	

### Cellular scale

On the micro-scale we employ a discrete cell-based model where each cell is represented by a set of variables that uniquely characterize it (Harjanto 2010). These are: the location, the state of each cell (proliferating, quiescent and necrotic) and the number of mitoses that separate it from the original cancer cell that gave rise to the tumor. At this scale our proposed model extends to the analogous models that are found in literature, by introducing a health level function h. This function represents the overall condition of each cell and is correlated with/to the equivalent amount of Adenosine 5-triphosphate (ATP) molecules of that each cell has got (Knowles 1980).

This is a fundamental concept of the proposed model which is explicitly justified as follows. Adenosine triphosphate is a nucleoside triphosphate used in cells as a co-enzyme. According to Knowles (1980) ATP is the ‘molecular unit of currency’ of intracellular energy transfer. While Campbell states that ‘ATP transports chemical energy within cells for metabolism. It is one of the end products of photophosphorylation, cellular respiration, and fermentation and is used by enzymes and structural proteins in many cellular processes, such as biosynthetic reactions, motility, and cell division’ (Neil et al. 2004). Specifically, a molecule of ATP contains three phosphate groups, and it is produced by a wide variety of enzymes, e.g. ATP synthase, from adenosine diphosphate (ADP) or adenosine monophosphate (AMP) along with various phosphate group donors. Substrate level phosphorylation, oxidative phosphorylation in cellular respiration, and photophosphorylation in photosynthesis are the three major mechanisms of ATP biosynthesis. Metabolic processes that use ATP as energy source convert it back to its precursors. Therefore ATP is continuously recycled in organisms. ATP is used by cells ‘as a substrate in signal transduction pathways by kinases that phosphorylate proteins and lipids, as well as by adenylate cyclase, which uses ATP to produce the second messenger molecule cyclic AMP. The ratio of ATP over AMP used as a way for a cell to sense how much energy is available, and control the metabolic pathways that produce and consume ATP’ (Hardie and Hawley 2001). According to Warburg et al. (1956; Hardie and Hawley 2001; Moses 1962) the rate of respiration in cancer cells is within an order of accuracy, identical to that of normal cells, while the glucose uptake is approximately 10 times higher than that of a normal cell. Furthermore, the complete combustion of glucose through the citric acid cycle and the electron-transport chain is at about 200 times decreased against the high rates of the anaerobic glycolysis process with lactic acid as an end product. All glucose molecules that are taken up, are primarily oxidized via respiration, while the remaining glucose molecules split to form lactic acid (Tiedemann 1952). Thus Warburg stated that although the anaerobic glycolysis yields only a fraction of the necessary cell energy (2 ATP), compared to the complete combustion of glucose (32 ATP). This relatively inefficient metabolic pathway is extremely preferable by the rapidly growing tumor cells even in the presence of oxygen. Recent studies, based on a more accurate estimation of ATP yields, during the oxidative phosphorylation steps, show that the complete oxidation of glucose rarely produces the full potential of 32 ATPs while a value of 30 ATPs is a more accurate estimation (Hinkle et al. 1991). In cancer cells this process is taking place reversely, where the anaerobic glycolysis acts as the mainstream metabolic pathway of glucose, whereas the Krebs cycle and oxidative phosphorylation have a supportive role (Gatenby and Gillies 2004).

### Evaluation of parameters

In order to perform the following simulations, some more estimations and assumptions have to be made. These approximations are necessary since most of the medical and biological data that are available in the literature are qualitative ones. Model parameter extraction from biological data and estimations are presented in the following paragraphs.

#### Cell size

 In the following simulations we assume that the tumor consists of a number of eukaryotic cells. Literature states that eukaryotic cell size varies between 10 and 30 μm, thus the approximation of 20 μm was used for the diameter of the cells.

#### Metabolic rates

 In accordance to the principles of Warburg et al. we propose that the glucose absorbed by each cell, will follow both metabolic paths. The amount of glucose that can be aerobically metabolized will do so in the mitochondria abiding to the stoichiometric laws using six parts of oxygen and one part of glucose to produce 30 parts of ATPs and the remaining glucose will be converted to 2 parts of ATP per glucose. Combining the work of Warburg et al. to the cell’s metabolic rates experimentally produced by Jiang et al. based on EMT6/Ro mouse mammary tumor cell line (Jiang et al. 2005), specific rates at which the proposed biochemical species produced or consumed from the tumor cells are provided on Table 2. Including the aforementioned methodology we are able to derive a new set of metabolic rates for each state of cancer cells as shown in the following examples:

#### Example of proliferating cell

 18 mM/h/cm$^{3}$ glucose will aerobically metabolize using the 108 mM/h/cm$^{3}$ of oxygen and will produce 540 mM/h/cm$^{3}$ ATP. The remaining 144 mM/h/cm$^{3}$ of glucose will produce 252 mM/h/cm$^{3}$ ATPs. Thus the total amount of ATP produced in the proliferating cells is 828 mM/h/cm$^{3}$. In what follows, this value will be indicated as $H_{P}$.

#### Example of quiescent cells

8.3 mM/h/cm$^{3}$ of glucose will aerobically metabolize with the 50 mM/h/cm$^{3}$ of oxygen and produce 250 mM/h/cm$^{3}$ ATPs. The remaining 71.7 mM/h/cm$^{3}$ of glucose will produce 143.4 mM/h/cm$^{3}$ ATP. Thus the total amount of ATP produced in quiescent cells is 393.4 mM/h/cm$^{3}$. In what follows of the present manuscript his value will be indicated as $H_{Q}$.

This process can be described as a first order chain reaction of oxygen and glucose producing ATP.

10 $$\begin{aligned} Glucose + Oxygen \,\, \underrightarrow{{\text {aerobic}}} \,\, E_{1} + Glucose \,\, \underrightarrow{{\text {anaerobic}}} \,\, E_{2} \end{aligned}$$

where $[E_{1}]$ is the concentration of the produced ATP molecules during the aerobic combustion of glucose, and $[E_{2}]$ is the concentration of produced ATP molecules during the anaerobic metabolism of the remaining glucose.

### Health function

Presumably we introduce a function that describes the metabolic rates of each cell according to its own state, named ‘health function' $\delta h_{t}^{i}$ of cell ‘i' which is given by:

11 $$\begin{aligned} \delta h_{t}^{i}= \frac{1}{H_{Q}}\left(\underset{S_{i}}{\int }E_{1} + \underset{S_{i}}{\int }E_{2}\right) - D(t)\delta t \end{aligned}$$

where $h_{t}^{i}$ denotes the health level at time t of cell ‘i’, and *D*(*t*) is the normalized decay rate of cellular energy that over time is equal to the ATP production of a quiescent cell and $\delta t$ is the simulated time between two iterations. We consider that the aerobic metabolism takes according to which where glucose is burned at the mitochondria in presence of oxygen.

12 $$\begin{aligned} E_{1}\left( \underset{S_{i}}{\int }C_{O}(\vec {u},\delta t), \underset{S_{i}}{\int }C_{G}(\vec {u},\delta t)\right) : C_{G} \in \left[ 0,162\right] , C_{O} \in [0,108] \end{aligned}$$

The remaining of the absorbed glucose is metabolized anaerobically in the cell.

13 $$\begin{aligned} E_{2}\left( \underset{S_{i}}{\int }C_{G}(\vec {u},\delta t)\right) : C_{G} \in \left[ 0,162\right] \end{aligned}$$

Considering that the 393.4 mM/h/cm$^{3}$ ATP is the energy transaction requirement for a cell to remain quiescent, then the remaining 434.6 mM/h/cm$^{3}$ of the totally 828 mM/h/cm$^{3}$ produced ATP, are the energy transactions required to proliferate. Also 1 mM/h/cm$^{3}$ of waste is considered to be produced for every 3.5 mM/h/cm$^{3}$ of ATP. As nutrition supplies become available for each cell through the environment, each cell then adds or retracts from its previous health level. Hence the proposed normalized health function, dictates that a proliferating cell will undergo mitosis at the target health value of:

14 $$\begin{aligned} h_{mitosis}= \dfrac{ H_{P}}{H_{Q}} \end{aligned}$$

The health function identifies explicitly the influence of the concentration of biochemical factors on the future health state of each cell. In the case where the health level of a cell increases more than the threshold value, i.e. takes values greater than 1.5, the state of this cell changes to proliferating. On the contrary, in the case where the health level decreases bellow a critical value, i.e. takes values smaller than 0.5, then this cell is characterized as necrotic and an apoptosis mechanism is implemented in the proposed model that dissolves the cell after a time period. The health level is representing the in-vivo behavior of cells, where cells with high amount of ATP are able to use this intracellular energy to perform various metabolic tasks such as movement and mitosis, while other cells, that are low in ATP, restrict cellular functionality. Thus depending on the $h_{t}$ variable, the function carries information with respect to the metabolic rates of each cell.

#### Oxygen

 Hemoglobin ($\left[ HB\right]$ or Hgb), is the iron-containing oxygen transport metalloprotein in the red blood cells of all vertebrates (Maton 1993). Hemoglobin in the blood carries oxygen from the respiratory organs to the rest of the body where it releases the oxygen in order to burn nutrients and provide energy to power the functions of the cells. The hemoglobin concentration in blood of an adult female is 122–150  g/L Hutler et al. (2000). The molecular weight of hemoglobin is 64,450  g/mol. Hence we can transform the above hemoglobin concentration into mmol/L (or mM = milli Molar, 1 Molar = 1 mole/L) as 0.0019–0.0023 mM/cm$^{3}$ for an adult female. The hemoglobin concentration decreases as blood flows from large arteries to the various tissues. For example the cerebral-to-large vessel hematocrit ratio is 0.69 (Wyatt et al. 1990). Hence if the hemoglobin concentration in blood is assumed 0.002 mM/cm$^{3}$ in large vessels, then assuming a breast tissue to large vessel hematocrit ration of 0.8 the hemoglobin concentration in blood in the regions of the breast is: 0.0016 mM/cm$^{3}$. When hemoglobin is totally saturated, each hemoglobin molecule carries four oxygen molecules. In the breast regions however, the blood in arterioles is not 100% saturated. It is more likely that the blood saturation (due to hemoglobin) is between 0.7–0.8. Hence assuming that the blood saturation is 0.75, then the oxygen concentration in blood in the region of the breast would be: C_B_ = 0.0016 × 4 × 0.75 = 0.0048 mM/cm^3^. The Hill equation (Goutelle et al. 2008) relates $C_{B}$ to $C_{P}$ (oxygen concentration in plasma) as:

15 $$\begin{aligned} C_{B}={C}_{P}+\frac{\left[ HB\right] {P}_{O_{2}}}{1+\left( \frac{aP_{50}}{{C}_{P}}\right) ^{h}} \end{aligned}$$

where:

- $\left[ HB\right]$ is the tetra haemoglobin concentration in blood that is equal to $0.0048\, \text{mM/cm}^{3}$
- $P_{O_{2}}$ is the oxyphoric power of tetra haemoglobin and is equal to 4
- h is the Hill coefficient equal to 2.73 (Hill 1910)
- $P_{50}$ the value of $P_{O2}$ at which haemoglobin is 50% saturated equal to 26 mmHg
- $\alpha$ is the solubility coefficient equal to $1.39\times 10\times 10^{-3}\,\text{mmol.L}^{-1}.\text{(mmHg)}^{-1}$

The solution of the equation indicates that at $C_{B}=0.005175$ mM, the oxygen concentration in plasma is $C_{P}=0.00053$ mM. Thus the following simulations were made under the assumption that the Dirichlet boundary value for the concentration of oxygen at the ECM is equal to the value of $C_{P}$ or 0.00053 mM/cm$^{3}$.

#### Glucose

 In humans, the normal glucose concentration of extracellular fluid that is regulated by homeostasis is presumed to have a value of 0.005 mM/cm^3^.

#### Waste–growth and inhibitor factors

 For these biochemical species we assume that all the factors that are produced by the tumor cells are nullified when they are diluted through blood circulation inside the human body. Thus an assumption is made that the Dirichlet data for these species at the ECM are set equal to zero.

### Mitosis

Mitosis is the part of the normal cell cycle by which a mother cell is separated into two identical daughter cells. Further we assume that the possibility for each cell to undergo mitosis changes according to its health level and to the biochemical environment. The function that describes the probability for mitosis of a proliferating cancer cell is a result of the following stochastic process:

16 $$\begin{aligned} P_{mitosis}=f\left( h_{t},C_{GF},C_{GI}, r, \mu , N; M, \sigma ^{2} \right) \end{aligned}$$

17 $$\begin{aligned} P_{mitosis} = {\left\{ \begin{array}{ll} \left( h_{t}-1\right) M +\epsilon (t) &{} : N < 60 \\ 0 &{} : N \ge 60 \end{array}\right. } \end{aligned}$$

The value M is defined as follows:

18 $$\begin{aligned} M = r \left( 1+\mu \right) \left( 1+ C_{GF} -C_{GI}\right) \end{aligned}$$

where:

- $h_{t}$ is the health value of the mother cell at time t
- M corresponds to the mean probability of mitosis and it’s a function of $r, \mu , C_{GF}, C_{GI}$
- *r* corresponds to the average mitosis probability of a normal tissue cell with value of 0.0315 as described by Ramis-Conde et al. (2008)
- $\mu$ corresponds to the effect on mitosis of the number of phenotype mutations
- $\sigma$ is the standard deviation of mitosis probability
- $C_{GF}$ and $C_{GI}$ the concentrations for, growth factors and growth inhibitor factors respectively
- N corresponds to the number of generations that separate each cell from its original cancer cell
- $\epsilon (t)$ is the Gaussian noise term

Once a mother cell undergoes mitosis, the two progeny cells occupy the same space at that time iteration and they are able to move freely thereafter. Also the health factor value of mother cells is equally divided between the two daughters upon mitosis. During mitosis, the number of phenotype mutations for each daughter cell has a probability to increase. This variable is introduced in the model, in order to represent the evolution of the cancer in more aggressive forms. This process as it was described explicitly above has been motivated from the work of Andrerson et al. (2011) and it is considered to describe comprehensively the biological phenomenon of mitosis. In the mitosis mechanism we have implemented a value for the Hayflick limit. This upper limit, as it is found in biological literature is set at 60 generations (Hayflick and Moorhead 1961).

### Cellular adhesion

Cellular adhesion is employed in the proposed model by implementing the differential adhesion hypothesis (DAH) as postulated by Malcolm Steinberg (2007), to model the mechanism of adhesion of the cells in the same state. The theory of cell adhesion advanced, to explain the mechanism by which heterotypic cells in mixed aggregates sort out into isotypic territories. The DAH postulates that tissues are visco-elastic liquids, and as such possess measurable tissue surface tensions. These surface tensions have been determined for a variety of tissues, including embryonic tissues. The surface tensions correspond to the mutual sorting behavior: the tissue type with the higher surface tension will occupy an internal position with regard to a tissue with a lower surface tension. Differences in homo and heterotypic adhesion presumed to be adequate to account for the phenomenon without requiring cell type specific adhesion systems. According to DAH, cellular movement and assortment is governed by the spontaneous rearrangement of cells, in much the same way as a liquid to obtain a more thermodynamically stable equilibrium. This is achieved by maximizing the amount of energy that is utilized in adhering the cells together, which decreases the free energy available in the system.

As cells with similar strengths of surface adhesion bond to each other, bonding energy in the overall system increases, and interfacial free energy decreases causing the arrangement to be thermodynamically more stable. Liquids behave in a similar manner, but with molecules moving due to their kinetic energy instead of motile cells moving around due to a combination of their kinetics and active movement. This allows examples of tissue spatial relation to correspond to the behavior of liquids, such as one tissue spreading across another corresponding to oil spreading across water; the oil spreads crossways the water to minimize weak oil-water interactions and maximize stronger water-water and oil-oil interactions, the cells similarly sort themselves to be near other cells of similar adhesive strength and bond with them. Other tissue interactions that DAH offers an explanation for included tissue hierarchy, are tissues with weaker surface adhesion that surround tissue with stronger surface adhesion, the rounding of irregular cell masses to become spherical, and the cell sorting and construction of anatomical structures that occurs independent of the path taken. This DAH mechanism forces the cells of the tumor, especially the proliferating cells, to remain connected to the tumor and not roam freely inside the simulated area while reinforcing the creation of agglomerations of necrotic cells.

### Chemotaxis

Chemotaxis is described as the effect of nutrients and waste in cellular movement or taxis and has been well documented (2011). In the proposed model while quiescent and necrotic cells are unaffected by taxis, proliferating cells make effort to move following the vector of the nutrient-waste function $\vec {m}$ according to:

19 $$\begin{aligned} \vec {m}(u,t) = \alpha \vec {\delta C_{O}(u,t)} + \beta \vec {\delta C_{G}(u,t)} - \gamma \vec {\delta C_{W}(u,t)} ,\quad \alpha ,\beta ,\gamma \in \mathbb {R}^{+} \end{aligned}$$

Cellular mechanics are used in order to ensure that the simulations produce comprehensive results. The implementation of cellular mechanics in the following simulations is done with the use of the Open Tissue algorithms for incompressible body dynamics and collision (Erleben et al. 2005s). The proposed model includes a mechanical physics engine that ensures that the representation of cellular life and movement remains coherent at all times. The following assumptions were made in order to enforce a physical coherence of the simulations:

- all cells are considered to be incompressible bodies
- between two adjacent cells an adhesion force is assumed in the form of a spring. Each of these springs forces attraction between the cells equal to $F = -kd$, as defined by Hooke’s law, where k is a constant factor characteristic of the spring, and d is the distance between the two neighboring cells.
- for proliferating cells subjected to chemotaxis, an extra force is assumed moving the cell on a vector of the nutrient-waste function, give in (19)

The types of connections between two neighboring and adjacent cells with respect to order of magnitude are $k_{QQ}$, $k_{PQ}$ or $k_{QP}$ and $k_{PP}$. while connections between a necrotic and cells of other type are neglected.
